# Co-expression Network Analysis Reveals Novel Genes Underlying Alzheimer’s Disease Pathogenesis

**DOI:** 10.3389/fnagi.2020.605961

**Published:** 2020-11-26

**Authors:** Rui-ting Hu, Qian Yu, Shao-dan Zhou, Yi-xin Yin, Rui-guang Hu, Hai-peng Lu, Bang-li Hu

**Affiliations:** ^1^Department of Neurology, Minzu Hospital of Guangxi Zhuang Autonomous Region, Nanning, China; ^2^Department of Pharmacy, Affiliated Hospital of Guilin Medical University, Guilin, China; ^3^Department of Research, Guangxi Medical University Cancer Hospital, Nanning, China; ^4^Department of Pharmacy, Minzu Hospital of Guangxi Zhuang Autonomous Region, Nanning, China

**Keywords:** Alzeheimer’s disease, gene, expreesion, pathway, miRNA

## Abstract

**Background**: The pathogenesis of Alzheimer’s disease (AD) remains to be elucidated. This study aimed to identify the hub genes in AD pathogenesis and determine their functions and pathways.

**Methods**: A co-expression network for an AD gene dataset with 401 samples was constructed, and the AD status-related genes were screened. The hub genes of the network were identified and validated by an independent cohort. The functional pathways of hub genes were analyzed.

**Results**: The co-expression network revealed a module that related to the AD status, and 101 status-related genes were screened from the trait-related module. Gene enrichment analysis indicated that these status-related genes are involved in synaptic processes and pathways. Four hub genes (*ENO2*, *ELAVL4*, *SNAP91*, and *NEFM*) were identified from the module, and these hub genes all participated in AD-related pathways, but the associations of each gene with clinical features were variable. An independent dataset confirmed the different expression of hub genes between AD and controls.

**Conclusions**: Four novel genes associated with AD pathogenesis were identified and validated, which provided novel therapeutic targets for AD.

## Introduction

Alzheimer’s disease (AD) is a common chronic and progressive neurodegenerative disease in the aging population that is characterized by brain atrophy, progressive memory loss, and clinical manifestations of cognitive impairment (Madore et al., 2020). Despite the great advancement made in the treatment of AD, its pathogenesis remains largely unknown, and no cure is currently available (Arvanitakis et al., 2019), which poses a great socioeconomic burden to society. To date, many factors have been shown to be important contributors to the pathogenesis of AD, such as age, genetic variants, and environmental exposure. According to current evidence, aberrant expression of cytokines has been found to be a crucial factor in AD development (Veitch et al., 2019). Hence, unraveling the molecular basis of this disease is necessary to uncover the biology of AD and identify novel therapeutic targets.

Previous studies have identified several genes that participate in the pathogenesis of AD, such as *IFITM3* (Hur et al., 2020), *HDAC1* (Pao et al., 2020), and *PLA2G4E* (Pérez-González et al., 2020), which lead to neuronal apoptosis, amyloid-β deposition, and tau hyperphosphorylation, and finally result in neuronal loss and neurofibrillary tangle (NFT) formation. However, there are still many genes involved in AD pathology that remain to be elucidated. Recently, the weighted gene co-expression network analysis (WGCNA) has been widely used to identify clusters of co-expressed genes with highly correlated expression patterns based on the genetic profile of many diseases (Zhao et al., 2010). WGCNA has also been adopted to screen crucial modules and genes that are associated with AD pathogenesis, and several genes have been identified and validated (Pandey et al., 2019; Shi et al., 2020; Soleimani Zakeri et al., 2020). However, there were several limitations in those studies, such as the small sample sizes of the datasets, the use of differentially expressed genes instead of the original genes, or the use of blood rather than nervous system tissues. Therefore, the mechanisms underlying AD remain to be explored.

In this study, we conducted a comprehensive integrative analysis for a gene dataset with a large number of samples from the brain tissues of AD patients and normal controls. Our work aimed to construct a co-expression network for the genes using the WGCNA method and to screen the hub genes that were related to AD pathogenesis; then we validated the robustness of the expression of hub genes using an independent validated cohort. We also analyzed the pathways and clinical significance of the hub genes. Our results will provide valuable molecular information of the pathogenesis of AD and contribute to developing potential therapeutic targets for AD in future studies.

## Materials and Methods

### Dataset Selection

**Figure 1:**
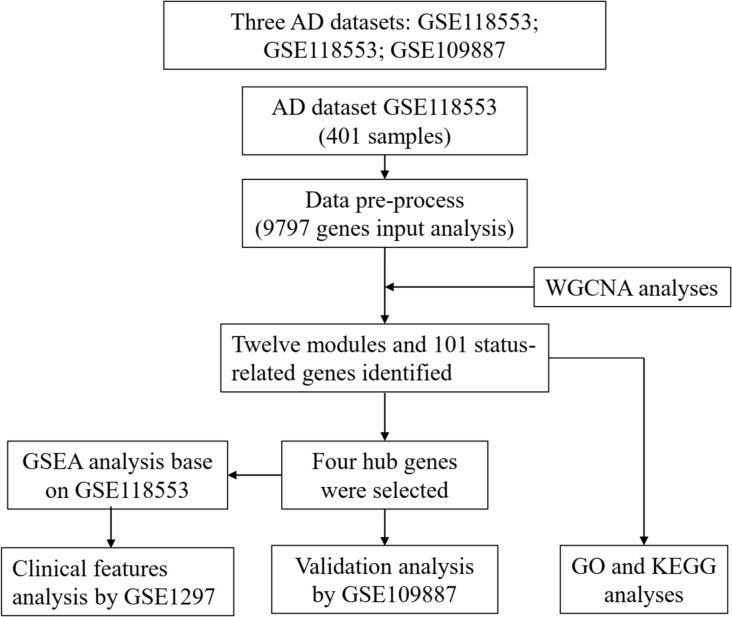
Flow chart of study design, data preparation, and analyses in this study.

A flow chart of study design, data preparation, and analyses in this study is shown in Figure 1. The human AD tissue mRNA expression data of the GSE118553 dataset (Patel et al., 2019) was downloaded from Gene Expression Omnibus (GEO) database. This dataset has 401 brain tissue samples, consisting of 167 AD tissues, 134 asymptomatic AD tissues, and 100 normal brain tissues. Individuals with intact cognition but neuropathology consistent with AD were diagnosed with asymptomatic AD (Patel et al., 2019). The brain tissues include the cerebellum, entorhinal cortex, frontal cortex, and temporal cortex. The platform of this dataset was GPL10558 (Illumina HumanHT-12 V4.0 expression beadchip). We also downloaded the GSE109887 dataset (Lardenoije et al., 2019), which has 40 AD tissues (middle temporal gyrus) and 32 normal brain tissues, as an independent validated dataset, and the platform was GPL10904. For the GSE1297 dataset (Blalock et al., 2004) with 22 AD tissues (middle temporal gyrus) and nine normal brain tissues, we used the GPL96 platform (Affymetrix Human Genome U133A Array) to analyze the clinical significance of hub genes.

### Data Preprocessing for the Selected Datasets

After the expression matrixes of the four GEO datasets were downloaded, the probes of each dataset were mapped to gene symbols using the corresponding platform. Probes with more than one gene or empty probes were removed. If there were numerous probes mapped to the same gene symbol, their mean value was selected as the gene expression value. For the GSE118553 dataset, which was used to conduct co-expression analysis, there are 47,325 probes in the Illumina array platform; after the duplicated and empty probe gene pairs were removed, 20,764 probe–symbol pairs remained. To ensure the robustness of the network construction, we selected the genes for which the mean expression was only more than 1/5 of the average expression.

### Weighted Gene Co-expression Network Analysis for Alzheimer’s Disease Dataset

WGCNA was performed on the GSE118553 dataset to identify significant modules and genes that were associated with the pathogenesis of AD. First, the soft-thresholding (β value) was set based on scale-free topology criterion to construct a correlation adjacency matrix. Then, the dynamic tree cut method was used to identify different modules. During module selection, the adjacency matrix was converted to a topology overlap matrix (TOM), and modules were detected by cluster analysis. Sample clustering was performed to analyze the relationship between gene expression and clinical features. The trait-related genes were extracted from the module that had significant correlations with clinical features and with high within-module connectivity. The WGCNA was implemented using R-Studio (version 3.4.2) software.

### Gene Function Enrichment Analysis

Gene Ontology (GO) and Kyoto Encyclopedia of Genes and Genomes (KEGG) pathway analyses were used to analyze the function involving the status-related genes. GO analyses included biological process (BP), molecular function (MF), and cellular component (CC). KEGG analysis was used to identify the significant pathways in which genes were enriched. The “clusterProfiler” package (Yu et al., 2012) was used to conduct the GO and KEGG analyses. *p*-value < 0.05 was considered statistically significant enrichment.

### Protein-Protein Interaction Network Construction and Hub Gene Selection

The trait-related genes were selected from the specific modules based on the results of WGCNA. In this study, we selected the genes significantly associated with the status of AD as interesting genes and named them as status-related genes. The status-related genes were input to the STRING online tool to construct a protein–protein interaction (PPI) network, and then the network was visualized using Cytoscape software. The hub genes of the status-related genes were further screened and visualized using cytoHubba plugin (Chin et al., 2014) of Cytoscape software.

### Gene Set Enrichment Analysis for the Hub Genes

Gene Set Enrichment Analysis (GSEA) method determines whether the pathways were randomly distributed at the top or bottom of the detected genes. We used the GSE118553 dataset as background gene sets to perform GSEA for each hub gene. Pathways were considered statistically significant with levels of adjusted *p*-value < 0.05. The “clusterProfiler” package (Yu et al., 2012) was used to conduct the GSEA.

### Correlation Analysis of Hub Genes With Clinical Features

The clinical information in the GSE1297 dataset included the stage of the disease, NFT value, Braak stage, Mini-Mental State Examination (MMSE) score, sex, and age. The associations of hub genes with clinical features were analyzed using Student’s *t*-test, ANOVA test, or Pearson’s correlation analysis in R-Studio software.

## Results

### Identification Hub Genes Associated With Alzheimer’s Disease Using Weighted Gene Co-expression Network Analysis

**Figure 2:**
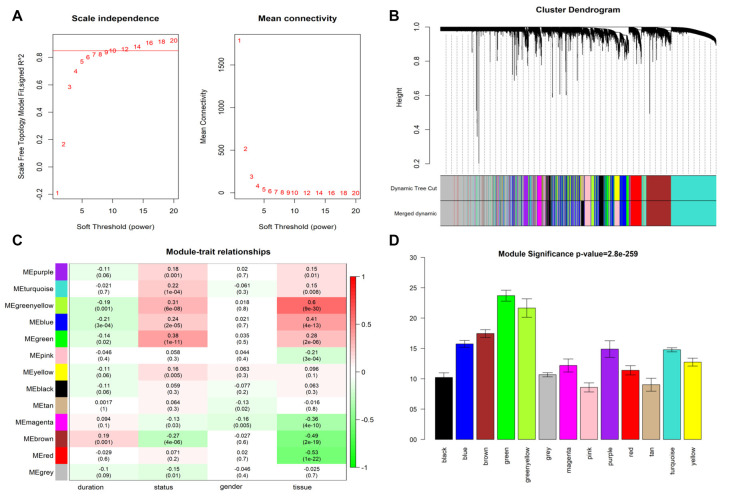
Weighted gene co-expression network analysis (WGCNA) for the GSE118553 dataset. **(A)** The soft-thresholding powers and the scale-free fit index. **(B)** Dynamic dendrogram of all genes clustered based on a dissimilarity measure. **(C)** Heatmap of the correlation between module eigengenes and clinical features of Alzheimer’s disease (AD). **(D)** Distribution of gene significance in the modules associated with status of AD.

After data preprocessing, 9,797 normalized genes profiled from the GSE118553 dataset with 401 samples (including AD, asymptomatic AD, and controls) were incorporated into the WGCNA method. The soft-thresholding power was set as 10 with the scale free index R^2^ as 0.85 (Figure 2A). Next, the modules were constructed using a dynamic tree-cut algorithm, and 12 modules were established (Figure 2B). Then, by integrating the clinical features with the modules, the relationship of clinical features with modules was presented, and the green module was mostly associated with the status of AD (Figures 2C,D). Thus, the genes in this module were selected, and the status-related genes were screened based on the selection criteria (selected criteria were set as the module membership >0.8 and the gene significance >0.2). Finally, we obtained 101 status-related genes that significantly associated with AD. Therefore, these status-related genes were used in the subsequent analyses.

### Functional Enrichment Analysis for Trait-Related Genes

**Figure 3:**
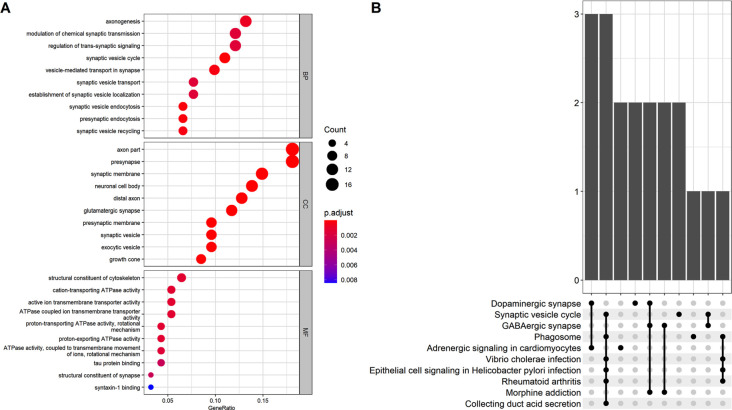
**(A)** Gene Ontology (GO) analysis for the status-related genes. **(B)** Kyoto Encyclopedia of Genes and Genomes (KEGG) analysis for the status-related genes.

The GO analysis revealed that the 101 AD status-related genes were mainly involved in the BP of axonogenesis, modulation of chemical synaptic transmission, and regulation of trans-synaptic signaling. The CC component enriched for axon, presynapse, and synaptic membrane genes, and the MF revealed structural constituents of the cytoskeleton, cation-transporting ATPase activity, and active ion transmembrane transporter activity genes. The KEGG pathway analysis revealed that these genes mainly involved the dopaminergic synapse, synaptic vesicle cycle, and GABAergic synapse (Figure 3).

### Selection of Hub Genes From Trait-Related Genes Using Protein-Protein Interaction Network Analysis

**Figure 4:**
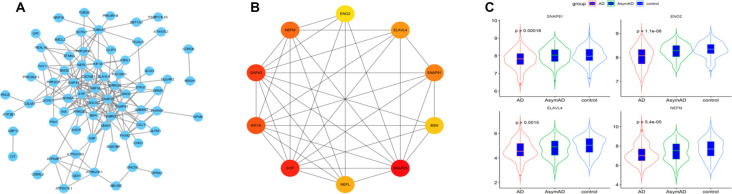
**(A)** The protein-protein interaction (PPI) network for the status-related genes. **(B)** Ten hub genes extracted from PPI network by cytoHubba plus-in of Cytoscape. **(C)** Expression of four hub genes among Alzheimer’s disease (AD), asymptomatic AD, and normal bran tissues.

The 101 AD status-related genes were used to construct a PPI network based on analysis using the STRING online tool. Then the PPI network was visualized by Cytoscape software, and the hub genes of the network were screened using the cytoHubba plug-in. Ten hub genes were identified, including *SNAP25*, *ENO2*, *ELAVL4*, *GAP43*, *SNAP91*, *SYP*, *BSN*, *NEFM*, and *NEFL* (Figures 4A,B). Among them, six genes (*SNAP25*, *KIF1A*, *GAP43*, *BSN*, *SYP*, and *NEFL*) have been widely investigated in AD in previous studies (Tien et al., 2011; Agostini et al., 2019; Jia et al., 2020; Ren et al., 2020; Wang M. et al., 2020). Therefore, we selected the remaining four hub genes (*ENO2*, *ELAVL4*, *SNAP91*, and *NEFM*) to analyze their role in AD. The expression of the four genes is shown in Figure 4C.

### Analysis of Clinical Significance of Hub Genes in Alzheimer’s Disease Using Another Independent Dataset

**Figure 5:**
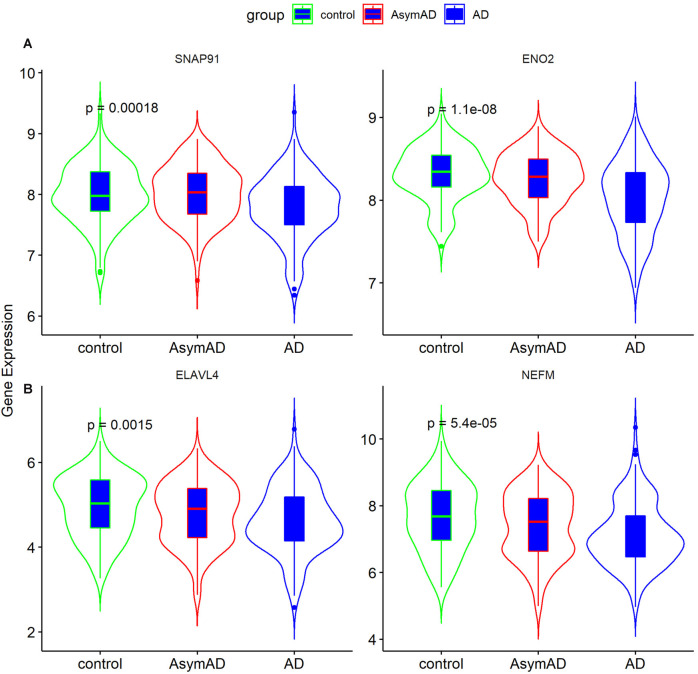
Expression of *ENO2*, *ELAVL4*, *SNAP91*, and *NEFM* in Alzheimer’s disease (AD) tissues. **(A)** Gene expression in different status (*n* = 31; ANOVA test). **(B)** Gene expression in different gender (*n* = 31; Student’s *t*-test). Data was expressed as mean ± SD, with *p*-value <0.05 as statistical significance.

The clinical features of the GSE1297 dataset were extracted. To examine the associations of hub genes with the clinical features, we first compared the gene expression of different sexes with status. The results showed that *NEFM* was differentially expressed in different status of AD; namely, *ENO2* and *ELAVL4* were differentially expressed in different sexes of AD patients, while the expression of remaining genes showed no significant difference in these clinical features (Figure 5). The correlations between hub genes and clinical phenotype were calculated by Pearson’s correlation analysis. As Table 1 shows, only NEFM was remarkably correlated to the age of AD patients (*p* < 0.05), while the other genes have no significant correlation with the NFT score, Braak stage, MMSE score, age, and PMI score (*p* > 0.05).

**Table 1:** Correlations of hub genes with the clinical features (*p*-value).

	*ENO2*	*SNAP91*	*NEFM*	*ELAVL4*
Braak	0.467	0.220	0.388	0.458
Age	0.061	0.414	0.035	0.295
MMSE	0.402	0.085	0.779	0.665
NFT	0.544	0.074	0.319	0.070

*Note. MMSE, Mini-Mental State Examination; neurofibrillary tangle*.

### Validation of Hub Genes in Alzheimer’s Disease Using Another Independent Dataset

**Figure 6:**
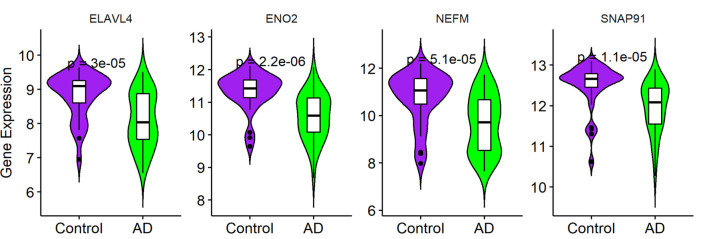
Validation of four hub genes in Alzheimer’s disease (AD) using GSE109887 dataset (*n* = 72; Student’s *t*-test). Data are expressed as mean ± SD, with *p*-value <0.05 as statistical significance.

To validate the expression of the four hub genes with AD, we used another microarray dataset (GSE109887), which included 40 AD and 32 normal tissues. The results revealed that the four hub genes in AD tissues were notably down-regulated compared with normal tissues in this validated dataset, demonstrating that these hub genes were all involved in the pathogenesis of AD (Figure 6).

### Gene Set Enrichment Analysis for the Hub Genes

**Figure 7:**
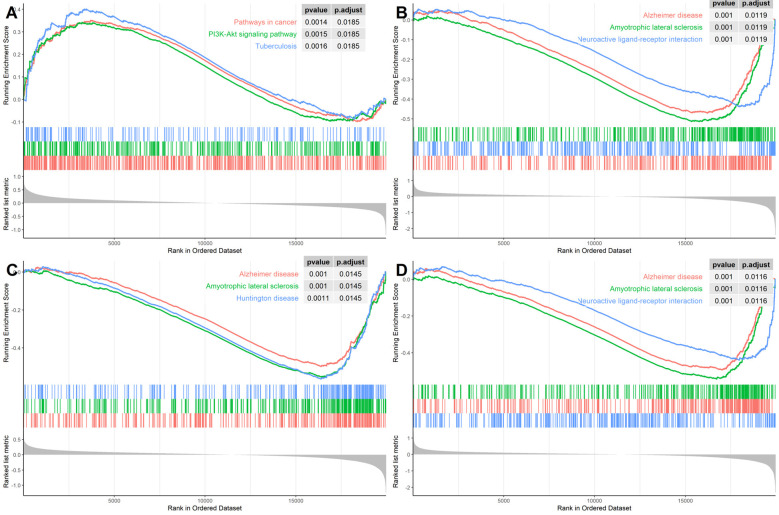
Gene Set Enrichment Analysis (GSEA) for the hub genes using GSE118553 dataset for **(A)** *ENO2*, **(B)** *SNAP91*, **(C)** *NEFM*, and **(D)** *ELAVL4*.

In order to identify the specific pathways that each of the four hub genes were involved in, we conducted the GSEA using the GSE118553 dataset as the background gene set. As Figure 7 illustrates, *SNAP91*, *NEFM*, and *ELAVL4* were all involved in AD pathways; *ENO2* was involved in a cancer pathway and the PI3K–AKT pathway; the latter pathway has also been reported in AD (Wang C. et al., 2020). These results confirmed the significant association of the four genes with AD.

## Discussion

In the present study, we performed comprehensive analyses on an AD dataset with a larger sample size. We constructed the gene co-expression network underlying AD pathogenesis and identified several gene modules clustered in this co-expression network. We screened the genes that were related to the clinical features and identified 101 genes that were remarkably related to the status of AD. By using Cytoscape and its plug-in, 10 hub genes were identified. We then analyzed four hub genes regarding which there is little knowledge in AD. The pathways of the four hub genes involved were determined, and the results indicated that these genes were all associated with AD pathways. Then, these hub genes were validated using an independent AD cohort, and the clinical feature analyses clarified the associations of these genes with AD.

Previous studies have discovered several pathways related to AD pathogenesis. For example, Wang and Wang (2020) analyzed differentially expressed genes in brain tissues and blood of AD patients compared with corresponding healthy individuals, and they found that MAPK and Wnt signaling pathways were significantly enriched in the hippocampus, temporal gyrus, and frontal gyrus. Sun et al. (2019) revealed that cancer-related and apoptosis pathways were considerably associated with AD pathogenesis. In the present study, we identified that the BPs of 101 AD status-related genes were enriched in synaptic-related signaling pathways, which is in agreement with our knowledge of the etiology of AD that synaptic failure is the pathological basis of cognitive impairment, the cardinal sign of AD (Selkoe, 2002). These results also confirmed the reliability of our study.

Among the four hub genes (*ENO2*, *ELAVL4*, *SNAP91*, and *NEFM*), although their function has been documented in other diseases, little is known regarding their role in AD. Enolase 2 (ENO2) has been found in mature neurons and is linked to brain iron accumulation-associated neurodegeneration (Takano et al., 2016). Previously, evidence has shown that the *ENO2* gene promoter drives high-level transgene expression in differentiated neurons throughout the central nervous system of transgenic zebrafish (Bai et al., 2007). Friedreich’s ataxia (FA) is a neurodegenerative disease, and a recent study showed that ENO2 is a marker of mitochondrial function and/or myelination status in FA patients (McMackin et al., 2019). In addition, expression of ENO2 was reportedly linked to prognosis for several cancers, including colorectal cancer (Pan et al., 2020), lung cancer (Liu et al., 2020), and pancreatic cancer (Zheng et al., 2020). These results demonstrated that ENO2 is involved in several diseases, and its role in AD warrants further exploration.

ELAV-like RNA binding protein 4 (ELAVL4) has been found to interact with other transcripts linked to AD, such as *APP* and *β-site APP-cleaving enzyme 1* (*BACE1*), and to increase the half-lives of these mRNAs (Kang et al., 2014). Expression of ELAVL4 protein was increased in mutant motor neurons and co-localized with mutant FUS in cytoplasmic speckles with altered biomechanical properties (De Santis et al., 2019). As one of the downstream targets of protein kinase C (PKC), ELAVL4 could modulate the stability and translation of specific target mRNAs involved in synaptic remodeling linked to cognitive processes (Talman et al., 2016). Taken together, ELAVL4 is linked to the pathogenesis of neurodegenerative diseases, including AD, and could possibly be a therapeutic target for AD.

Synaptosome associated protein 91 (SNAP91), also known as AP180, has been shown to be significantly increased in schizophrenia compared with normal controls (Fromer et al., 2016). In an integrated analysis of whole exome sequencing and copy number evaluation in Parkinson’s disease (PD), loss of function and missense changes in SNAP91 were observed in PD patients (Yemni et al., 2019). SNAP91 was also found to promote release site clearance and clathrin-dependent vesicle reformation in mouse cochlear inner hair cells (Kroll et al., 2020).

Neurofilament medium (NEFM) is relevant to the elongation of neuronal structures (Pezzini et al., 2017). In a larger cohort with 367 amyotrophic lateral sclerosis (ALS) patients and 101 controls, plasma NEFM levels were significantly elevated in ALS patients compared with controls (Häggmark et al., 2014). NEFM was also down-regulated in ZF-like aldosterone-producing adenomas and contributed to a D1R/D2R imbalance (Maniero et al., 2017). The above evidence indicates an association of both SNAP91 and NEFM with neurodegenerative diseases; however, their association with AD has not been reported previously, and their roles in AD still require further investigation.

In this study, we analyzed the pathways of four hub genes and the results indicated that these pathways were associated with AD or other neurodegenerative diseases, demonstrating their key roles in these diseases. In the validation analysis, the four hub genes all showed significantly different expression between AD and normal brain tissues, suggesting the robustness of our results. However, in the analysis of hub genes with the clinical features, we found that the associations were varied, indicating the different roles of these genes in the development of AD. We speculate that these results might be due to the small sample size and different brain tissues (middle temporal gyrus) of the GSE1297 dataset compared with the GSE118553 dataset; hence, these results still need to be validated in a larger sample size and using the same brain tissues.

There are several limitations to this study. First, the GSE118553 dataset contains data from two brain region tissues (cerebellum and cortex); although these two tissues are involved in the pathogenesis of AD, the robustness of our results was reduced due to the mixture of data. Second, the tissues of the validated cohort were different from those of the initial analysis; thus, the results need to be validated in another cohort using the same tissues. Third, since this study was a bioinformatics analysis, the roles of ENO2, ELAVL4, SNAP91, and NEFM need to be validated in *in vivo* and *in vitro* experiments.

## Conclusion

The present study identified the novel association of four genes (*ENO2*, *ELAVL4*, *SNAP91*, and *NEFM*) with AD pathogenesis by using gene co-expression network analysis based on the clinical and pathological status of disease. The roles the four genes were also identified. These results highlight the molecular mechanism underlying AD and will assist in finding novel therapeutic targets for AD.

## Data Availability Statement

The original contributions presented in the study are included in the article, further inquiries can be directed to the corresponding author/s.

## Author Contributions

Study concept and design were done by B-lH and R-tH. Collection and assembly of data were performed by QY, Y-xY, and S-dZ. Data analysis and interpretation were carried out by QY, Y-xY, R-gH, and S-dZ. B-lH and H-pL revised the manuscript. All authors contributed to the article and approved the submitted version.

## Conflict of Interest

The authors declare that the research was conducted in the absence of any commercial or financial relationships that could be construed as a potential conflict of interest.

## Abbreviations

AD: Alzheimer’s disease
WGCNA: Weighted Gene Co-expression Network Analysis
ENO2: enolase 2
ELAVL4: ELAV-like RNA binding protein 4
SNAP91: synaptosome associated protein 91
NEFM: neurofilament medium.
